# miR-29b-3p Increases Radiosensitivity in Stemness Cancer Cells *via* Modulating Oncogenes Axis

**DOI:** 10.3389/fcell.2021.741074

**Published:** 2021-09-16

**Authors:** Dong Pan, Yarong Du, Rong Li, Aihua Shen, Xiaodong Liu, Chuanyuan Li, Burong Hu

**Affiliations:** ^1^Department of Radiation Medicine, School of Public Health and Management, Wenzhou Medical University, Wenzhou, China; ^2^Key Laboratory of Heavy Ion Radiation Biology and Medicine of Chinese Academy of Sciences, Key Laboratory of Space Radiobiology of Gansu Province, Institute of Modern Physics, Chinese Academy of Sciences (CAS), Lanzhou, China; ^3^Department of Dermatology, Duke University Medical Center, Durham, NC, United States

**Keywords:** miR-29b-3p, three dimensional cultured cells, stemness, oncogene axis, radiosensitivity

## Abstract

Radioresistance conferred by cancer stem cells (CSCs) is the principal cause of the failure of cancer radiotherapy. Eradication of CSCs is a prime therapeutic target and a requirement for effective radiotherapy. Three dimensional (3D) cell-cultured model could mimic the morphology of cells *in vivo* and induce CSC properties. Emerging evidence suggests that microRNAs (miRNAs) play crucial roles in the regulation of radiosensitivity in cancers. In this study, we aim to investigate the effects of miRNAs on the radiosensitivity of 3D cultured stem-like cells. Using miRNA microarray analysis in 2D and 3D cell culture models, we found that the expression of miR-29b-3p was downregulated in 3D cultured A549 and MCF7 cells compared with monolayer (2D) cells. Clinic data analysis from The Cancer Genome Atlas database exhibited that miR-29b-3p high expression showed significant advantages in lung adenocarcinoma and breast invasive carcinoma patients’ prognosis. The subsequent experiments proved that miR-29b-3p overexpression decreased the radioresistance of cells in 3D culture and tumors *in vivo* through interfering kinetics process of DNA damage repair and inhibiting oncogenes RBL1, PIK3R1, AKT2, and Bcl-2. In addition, miR-29b-3p knockdown enhanced cancer cells invasion and migration capability. MiR-29b-3p overexpression decreased the stemness of 3D cultured cells. In conclusion, our results demonstrate that miR-29b-3p could be a sensitizer of radiation killing in CSC-like cells *via* inhibiting oncogenes expression. MiR-29b-3p could be a novel therapeutic candidate target for radiotherapy.

## Introduction

With the development of advanced radiotherapy techniques, radiotherapy has become an extremely efficacious treatment for cancers (Govindan et al., 2009). However, radioresistance remains a major obstacle for cancer treatment and represents the main reason for radiotherapy failure, which is associated with poor prognosis in cancer patients and can ultimately lead to tumor recurrence and metastases (Tang et al., 2018). A newly emerged plausible explanation for tumor radioresistance is the existence of a subpopulation of cancer stem cells (CSCs) that are intrinsically more resistant to multiple clinical therapies. CSCs are able to self-renew and differentiate and possess a high capability to repair DNA damage, exhibit low levels of reactive oxygen species, and proliferate rapidly. These features render CSCs resistant to various therapies, including radiotherapy (Phillips et al., 2006; Blazek et al., 2007; de Jong et al., 2010; Li et al., 2016). Thus, exploring the mechanism of radioresistance in CSCs would provide therapeutic targets to sensitize CSCs to cytotoxic therapies and improve the efficacy of radiotherapy.

Three dimensional (3D) cultured model *in vitro* is an approach that fulfills a need for contemporary cancer research and drug resistance studies. It could induce the formation of organoid tissue, such as embryonic lung and salivary gland epithelial cells that can aggregate and form branched organoids in an appropriate 3D microenvironment (Wei et al., 2007). In addition, the 3D cultured microenvironment enhances CSC properties of cancer cells, which is a useful platform for anticancer therapeutics and CSC research (Chen et al., 2012; Xue et al., 2015b; Usui et al., 2016).

Matrigel is commonly used for the establishment of a 3D cell culture model *in vitro.* Matrigel is a gelatinous protein mixture derived from Engelbreth–Holm–Swarm mouse sarcoma cells (Roskelley et al., 1994). Compared with the traditional two-dimensional (2D) monolayer culture, 3D cell culture could mimic the morphology of cells *in vivo* (Le Beyec et al., 2007; Lelièvre, 2009). In addition, the response behaviors of cells in 2D and 3D cultures for stress are different. Cancer cells in 3D culture are more chemoresistant and radioresistant compared with 2D culture (Hehlgans et al., 2009; Storch et al., 2010). Our previous study showed that the 3D growth microenvironment in matrigel impacts epigenetic regulation, including DNA methylation and reprogramming, which are responsible for radioresistance (Xue et al., 2015b; Pan et al., 2016a). However, the reason behind the difference in radioresistance between 2D- and 3D-grown cancer cells remains largely unclear.

MicroRNAs (miRNAs) are a class of endogenous small non-coding ribonucleic acid molecules that are involved in the regulation of gene expression. After transcription and cleavage in the nucleus, mature miRNAs are loaded onto the RNA-induced silencing complex, where they can bind to a specific seed sequence in the 3′ untranslated region of target genes and promote degradation of messenger RNAs. They are involved in many physiological and pathological processes, including cell proliferation, apoptosis, development, and carcinogenesis (Lagos-Quintana et al., 2001; Bartel, 2004). The functions of miRNAs are recently being appreciated for their important roles in response to radiotherapy through regulating genes expression (Czochor and Glazer, 2014). Our previous studies indicated that miR-142-3p is involved in radiation-induced premature chromatid separation, and miR-145 regulates cell responses to irradiation (Xue et al., 2015a; Pan et al., 2016b). Understanding the regulation and function of miRNAs is essential to improving current cancer therapy.

In this study, the role of miRNA in radioresistance was investigated. Using miRNA microarray analysis in 2D and 3D cell culture models, it was found that miR-29b-3p and its targeted oncogenes were differentially expressed in 3D cultured A549 and MCF7 cells compared with 2D cultured cells. Clinical data analysis showed that miR-29b-3p contributes to the poor prognosis of lung adenocarcinoma (LUAD) and breast invasive carcinoma (BRCA) patients. Meanwhile, miR-29b-3p overexpression enhanced the radiosensitivity of 3D cultured cells and tumors *in vivo via* impeding the DNA repair dynamic process and weakening the invasion and migration capacity. Knocking down of miR-29b-3p was associated with low expression of oncogenes in cancer cells, particularly in CD133^+^ CSCs. Our findings suggested that miR-29b-3p enhances radioresistance *via* regulating oncogenes in cancer cells.

## Materials and Methods

### Cell Culture

A549 (human lung carcinoma), MCF7 (human breast carcinoma), and LLC1 (mouse lung carcinoma) cells were obtained from the American Type Culture Collection (Manassas, VA, United States). For monolayer cells culture, A549 cells were cultured in RPMI-1640 medium (Gibco, United States), and MCF7 and LLC1 cells were cultured in high-glucose Dulbecco’s modified Eagle’s medium (Gibco), supplemented with 10% fetal bovine serum (Hyclone, United States) and 1% penicillin/streptomycin (Amresco, United States). Construction of 3D cultured microenvironment using matrigel matrix (BD, United States) was performed as described previously (Asaithamby et al., 2011). Briefly, 30-μl trypsinized cells at a density of 1.5 × 10^6^ cells/ml were mixed with 250-μl pre-thawed matrigel and seeded into a single well of 12-well tissue culture plates. Two 2-ml media were added after incubating for 30 min at 37°C. All 3D-grown cells were cultured in matrigel for 5 days before subsequent experiments. The medium was changed every 2 days. Both 2D- and 3D-grown cells were cultured at 37°C in a humidified atmosphere containing 5% carbon dioxide.

### Radiation

X-ray irradiation was carried out with a Faxitron RX-650 facility (Faxitron Bioptics, United States) at a dose rate of 0.75 Gy/min, which was operated at 100 kVp 5 mA at room temperature. The dose rate was measured using the Ray meter (RX-650, Germany).

### MicroRNA Microarray Analysis

Samples of 2D and 3D cultured A549 cells were collected. Then, the microarray analysis of miRNAs was performed by CapitalBio Corporation following the protocol as previously described (Guo et al., 2008). In brief, all of the miRNA probe sequences were designed to be fully complementary to their cognate mature miRNA. Oligonucleotide probes were synthesized and were printed in triplicate using the SmartArraymicroarrayer (CapitalBio). Total RNAs were isolated from these cells with TRIzol reagent (Invitrogen, United States) 0.5 h after 5-Gy X-ray irradiation. RNA was labeled using the T4 RNA ligase, and the hybridization was performed in a hybridization cassette. Gene Spring Software (Agilent, Santa Clara, CA, United States) was used for data analysis. Values at least two times higher than the background were screened out for analysis.

### DNA and RNA Extraction

Genomic DNA was extracted from the cells by using Wizard^®^ SV Genomic DNA Purification System (Promega, United States) according to the instruction. Extraction of total RNA from cells used for qRT-PCR was performed using the E.Z.N.A.^®^ Total RNA Kit (Omega, United States) following the manufacturer’s protocol.

### Quantitative Real-Time Polymerase Chain Reaction

Reverse transcription was conducted with the ALL-in-one^TM^ miRNA quantitative real-time polymerase chain reaction (qRT-PCR) detection kit (GeneCopoeia, United States). Primers for miR-29b-3p and the U6 internal control for humans and mice were purchased from GeneCopoeia (China). MiR-29b-3p-human (Ca: HmiRQP0373), miR-29b-3p-mice (Ca: MmiRQP0373), universal reverse primer, and U6 forward primer were from the kit. qRT-PCR was performed using a Bio-Rad Chromo4 System Real-Time PCR detector (Bio-Rad, United States). All procedures were conducted according to the manufacturers’ protocols under the following conditions: initiation for 10 min at 95°C, followed by 40 thermal cycles each at 95°C for 10 s, 60°C for 20 s, and 70°C for 10 s. Relative fold-change in miRNA expression was calculated using the 2^–ΔΔ*CT*^ method with the following equation: RQ (Relative Quantitation) = 2^–ΔΔ*Ct*^.

### Patients Clinical Data

All patients’ clinical data were obtained from the KM plotter database^1^, including multiple studies from Gene Expression Omnibus, The Cancer Genome Atlas, and Molecular Taxonomy of Breast Cancer International Consortium (Nagy et al., 2018). The analyzed cohort includes 504 LUADs and 1,262 BRCAs within the timeframe of our study (April 5, 2019 to June 10, 2020). For survival analysis, Kaplan–Meier survival curves were generated by using the statistical software GraphPad Prism. The non-parametric Mantel–Cox log-rank test was used to determine the statistical differences among different patient groups.

### Western Blot

Cells were lysed in radioimmunoprecipitation assay buffer (Beyotime, China) with Protease Inhibitor Cocktail Tablets (Roche, Switzerland). The total protein concentrations of the lysates were determined using the Bio-Rad protein assay kit. Equal amounts of protein were denatured with loading buffer (Beyotime) at 100°C for 10 min, then loaded in 10% sodium dodecyl sulfate–polyacrylamide gel electrophoresis for electrophoresis and transferred to a methanol-activated polyvinylidene fluoride membrane (Millipore, United States). The membrane was blocked in tris-buffered saline containing 5% bovine serum albumin (MP Biomedical, United States) for 1 h at room temperature. Primary antibodies were incubated overnight at 4°C. The primary antibodies included AKT2, CD133, DNMT3B, MYC, PIK3R1, RBL1 (1:1,000, Proteintech, United States), Bcl-2 (1:1,000, Affinity Biosciences, United States), and GAPDH (1:2,000, ZSGB-BIO, China). After washing with tris-buffered saline twice, the membrane was incubated with the appropriate horseradish peroxidase-labeled secondary antibody for 1 h at room temperature. The secondary antibody conjugated with horseradish peroxidase is goat anti-rabbit immunoglobulin G (1:5,000, ZSGB-BIO). Immunoblots were visualized using enhanced chemiluminescence detection system according to the manufacturer’s protocol.

### Cell Transfection

Si-miR-29b-3p (knockdown), Over-miR-29b-3p (overexpression), and negative control lentivirus were purchased from Genechm (China). For the construction of miR-29b-3p deficiency and overexpression stable cell lines, cells were plated on the day before the lentivirus infection at a confluence of 30–50%; the multiplicity of infection is 10. The medium was changed 24 h post-infection, and cells were selected for 7 days with 1 μg/ml puromycin.

### Dissociation of Three-Dimensional Structure

Three-dimensional cultured cells were recovered from matrigel using recovery solution (BD) according to the manufacturer’s instructions as described previously (Lee et al., 2007). In brief, matrigel containing the 3D structures was first washed with ice-cold PBS and then removed from the well. After being transferred to a 15-ml tube containing the pre-chilled recovery solution (1 ml per well), the mixture was incubated on ice for 45 min with intermittent mixing and then centrifuged at 1,000 rpm for 10 min at 4°C. The supernatant containing the dissolved matrigel was discarded, and the 3D structures were washed once with PBS. To make a single-cell suspension of recovered 3D structures, cells were trypsinized using trypsin- ethylenediaminetetraacetic acid (0.25%, Invitrogen). Dissociated cells were used for colony formation assay.

### Colony Formation Assay

Cells from 2D culture were washed with PBS buffer, trypsinized, and counted using a cell counter (Coulter) after irradiation. Cells from 3D culture converted to single-cell suspension as described earlier and resuspended in medium. An appropriate number of cells (0:100, 1:200, 2:500, 4:2,000, and 6:10,000)were seeded into each Φ60 dish in 5 ml of complete media. After 10 days of incubation, colonies were fixed with 10-ml fresh Carnoy’s fluid, stained with 0.5% crystal violet for 20 min. Colonies with more than 50 cells were recorded and counted manually under an inverted microscope. Plating efficiencies (PEs) were calculated as follows: numbers of colonies formed/numbers of cells plated. Cell surviving fractions were calculated as follows: PE (irradiated)/PE (unirradiated). All experiments were performed in triplicate.

### Promoter Methylation Analysis

The extracted genomic DNA was subjected to bisulfite modification using the EpiTect Fast DNA Bisulfite Kit (Qiagen) according to the manufacturer’s instruction. After that, the bisulfite-converted genomic DNA was amplified by a set of RBL1 primers for the unmethylated reaction and methylated reaction to the methylation-specific PCR as described previously (Pan et al., 2016a): Unmethylated forward primer (5′ GGAGGTATTTTATTATGTTGTATGA) and reverse primer (5′ TCCTTAACCCTTAACTAATCACAAA), methylated forward primer (5′ GGAGGTATTTTATTACGTTGTACGA), and reverse primer (5′ CTTAACCCTTAACTAATCGCGAA). PCR was carried out with MyCycler RCR (Bio-Rad) by using the following condition: 94°C for 5 min, followed by 40 cycles of 94°C for 30 s, 52°C (unmethylated primer) or 56°C (methylated primer) for 30 s, and 72°C for 30 s.

### Cell Invasion Assay

Cells (1 × 10^5^) were resuspended in 100 μl of serum-free medium and seeded in matrigel-coated transwell upper chambers (Millipore, United States) with 8.0 μm polycarbonate filter inserts in 24-well plates, whereas the bottom chambers were filled with 600-μl complete medium. After incubation for 24 h, non-migrated cells and the matrigel were scraped using a cotton swab. The bottom side of the membrane was fixed with ethanol and stained with Giemsa. The transwell chambers were washed three times with PBS. Images of migrated cells were obtained using a microscope (Carl Zeiss, Germany).

### Wound Healing Migration

Cells were trypsinized, and 1 × 10^5^ cells per well were added to the 12-well plate with complete media. When cells reached approximately 90% confluency, scratch wounds were created on monolayers by a sterile 200-μl micropipette tip. Then, cells were washed twice with a fresh medium to remove any loosely held cells. Photographs were taken immediately (0 h) and 24 h later. Images at 0 and 24 h were compared, and the area of the wound closure was calculated using Image J software.

### Immunocytochemical Staining

To detect 53BP1 and γ-H2AX foci that form at the DSB sites, 1 × 10^4^ cells were seeded on glass coverslips in each well of a 12-well plate, cultured for 24 h before radiation. Subsequent experimental procedures followed the previous description (van Oorschot et al., 2014). After the 1-Gy X-ray irradiation, cells were fixed with 4% paraformaldehyde for 20 min at room temperature and permeabilized with 0.5% Triton X-100 in PBS for 10 min. Non-specific binding sites were blocked with 5% bovine serum albumin (BSA) in PBS for 60 min at room temperature before probing with primary antibodies. The primary antibodies used for immunostaining include 53BP1 (1:3,000, Abcam, United States) and γ-H2AX (1:1,000, CST). Secondary antibodies (anti-mouse or rabbit conjugated with Alexa 488/633) were purchased from Beyotime (1:2,000). Then, the cells were incubated in the primary antibody diluted in 5% BSA/1 × PBS 1 h at room temperature. After incubation, cells were washed three times with PBS and then incubated with the appropriate Alexa Fluor secondary antibodies diluted in 5% BSA for 1 h. Cells were washed three times with PBS again. The nuclei were counterstained with 4′,6-diamidino-2-phenylindole (Beyotime). Digital image analysis was performed to determine the number of γH2AX and 53BP1 foci by fluorescence microscope (Axio Imager Z2) at 63 × magnification and confirmed by visual inspection of images. Quantification of foci per cell was carried out from images of 50 cells for every time point from at least three independent experiments.

### Tumor Irradiation in Mice

Animal experiments conducted in this study were approved by Wenzhou Medical University Institutional Animal Use and Care Committee. C57BL/6J mice were purchased from Beijing Vital River Laboratory Animal Technology Co., Ltd. Before tumor cell injection, age-matched 6-week-old female mice were shaved at the right hindlimbs. LLC1 cells (2 × 10^5^) were resuspended in 50-μl PBS and injected into shaved flanks subcutaneously with negative control or miR-29b-3p overexpression tumor cells. After 7 days, tumors were irradiated with a single 8-Gy dose of X-rays with the help of lead shielding. Tumor volumes were measured every other day and calculated by the formula: (length) × (width)^2^/2. The mice were killed when tumor volumes reached 2,000 mm^3^. Kaplan–Meier estimator and log-rank (Mantel–Cox) test were used for survival analysis among different tumor-bearing mice groups.

### Flow Cytometry Side Population Assessment and CD133^+^ Cell Sorting

Flow cytometry was used to identify lung cancer stem cells with positive CD133 (Ferrandina et al., 2009). After dissociation with trypsin and subsequent neutralization, 2 × 10^6^ A549 cells were resuspended in 500-μl PBS, and 20-μl monoclonal antibody CD133 (Miltenyi Biotec, United States) was added. The mixture was incubated for 20 min at room temperature and protected from light. After incubation, cells were washed twice with PBS and resuspended in a 500-μl medium. Background fluorescence was estimated by substituting the specific primary antibody with specific isotype controls. Samples were acquired on a FACSCanto II flow cytometer (BD Biosciences) and analyzed with FACSDiva software (BD Biosciences). The fresh isolated CD133 + cells were cultured before assay in a stem cell medium containing a serum-free Dulbecco’s modified Eagle’s medium/F12 (1:1) medium (Gibco-BRL), 20 ng/ml epidermal growth factor, 20 ng/ml basic fibroblast growth factor, and 20 ng/ml leukemia inhibitor factor (LIF) (all were from Miltenyi Biotec).

Side population assessment by Hoechst 33342 analysis followed the previous description (Tirino et al., 2009). Both 2D and 3D cultured A549 cells were trypsinized and suspended at 2 × 10^6^ cells/ml in a medium with 5 mg/ml Hoechst 33342 (Sigma, United States) for 90 min at 37°C with intermittent stirring. After incubation, the cells were washed in PBS and kept on ice for 5 min until analyzed by a flow cytometer FlowSight (Amnis, United States). The Hoechst 33342 dye was excited at 350-nm ultraviolet, and the resultant fluorescence was measured at two different wavelengths using 424/44 BP and 675 LP filters for detection of Hoechst blue and red, respectively. The results were analyzed using Flowjo software.

### Statistics

All experiments *in vitro* were performed in triplicate and repeated at least three times. Statistical significance (*P*-values) of differences in means between two samples were evaluated using unpaired *t*-tests. Data are represented as individual values or as mean ± standard error of the mean. Group sizes (n) and applied statistical tests are indicated in figure legends. For *in vivo* experiments and clinical data analysis, log-rank (Mantel–Cox) tests were used for survival analysis, and tumor volume significances were assessed by two-way analysis of variance among different groups. Statistics were calculated using GraphPad Prism 8.2.1. *P* < 0.05 was considered statistically significant (^∗^), *P* < 0.01 as highly significant (^∗∗^), and *P* < 0.001 (^∗∗∗^) as extremely significant.

## Results

### Low Expression of miR-29b-3p in A549 and MCF7 Cells in Three-Dimensional Culture Compared With Two-Dimensional Culture

**FIGURE 1:**
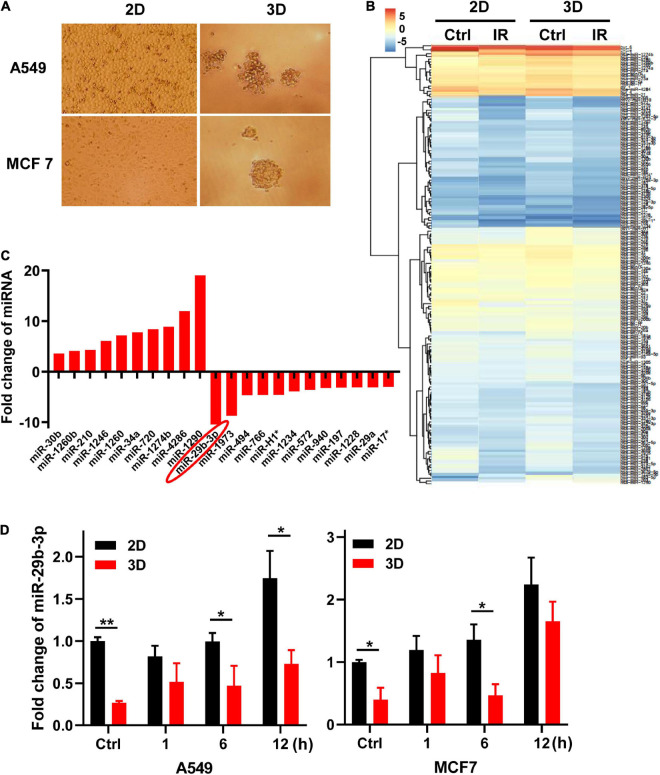
MiR-29b-3p expression in 2D and 3D cultured A549 and MCF7 cells. **(A)** The morphology of 2D and 3D cultured A549 and MCF7 cells captured under a phase-contrast microscope. **(B)** Heat map of miRNAs microarray analysis in 2D and 3D cultured A549 cells 0.5 h after 5 Gy X-ray radiation. **(C)** Fold changes of miRNAs expression that are more than threefold between 2D and 3D cultured cells. **(D)** Relative miR-29b-3p expression measured by qRT-PCR at indicated time points in 2D and 3D cultured A549 and MCF7 cells after 5 Gy X-ray irradiation. U6 was used as an internal control. Ctrl, unirradiated groups. Significance was determined by unpaired *t*-test. *P < 0.05; **P < 0.01.

The morphological features of A549 and MCF7 cells are different in 2D and 3D cultures (Figure 1A). Two-dimensional cultured cells were flat and formed a monolayer on the Petri dish, whereas cells in 3D culture in the matrigel formed microspheres. To identify the different characteristics of miRNAs in cells between 2D and 3D cultures, we analyzed the miRNA expression profiles of the 2D and 3D cell cultures of A549 cells 0.5 h after exposure to 0- and 5-Gy X-rays using miRNAs microarray analysis (Figure 1B). We found 10 miRNAs were upregulated, whereas 11 were downregulated with more than threefold changes between 2D and 3D cultures (Figure 1C). MiR-29b-3p had the biggest low fold change in 3D compared with 2D cultures (−10.43). For further investigation, we measured the expression of miR-29b-3p in A549 and MCF cells in the 2D and 3D cultures after 5-Gy X-ray irradiation by qRT-PCR. As shown in Figure 1D, the expression of miR-29b-3p was significantly lower in 3D culture compared with 2D culture both in irradiated and unirradiated cells. These results indicated that the miRNA, particularly miR-29b-3p, expression profiles in cells between 2D and 3D cultures are different.

### miR-29b-3p High Expression Is Associated With Better Prognosis of Lung Adenocarcinoma and Breast Invasive Carcinoma

**FIGURE 2:**
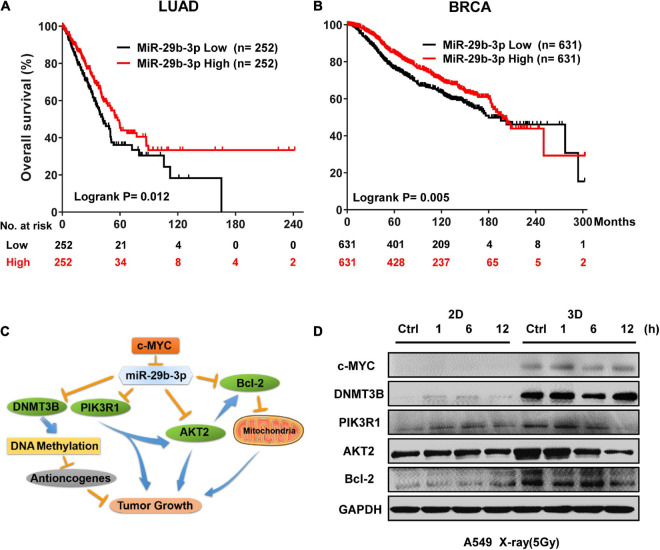
MiR-29b-3p expression affects survival probability in LUAD and BRCA by regulating oncogenes. **(A)** Kaplan–Meier survival curves of LUAD patients with low or high miR-29b-3p expression. **(B)** Kaplan–Meier survival curves of BRCA patients with low or high miR-29b-3p expression. **(C)** A schematic representation showing the signaling pathway of miR-29b-3p suppressing tumor growth by targeting a series of oncogenes. **(D)** Western blot assay on the expression of c-MYC, DNMT3B, PIK3R1, AKT2 and Bcl-2 at the indicated time points after 5 Gy X-rays in 2D and 3D cultured A549 cells. Ctrl, unirradiated groups. The cut-off values of high and low are median in LUAD and BRCA. *P*-values calculated by use of logrank test.

To explore the potential association between miR-29b-3p expression and overall survival of cancer patients, we analyzed the miR-29b-3p expression status and overall survival in LUAD and BRCA patients. Published clinical and genomic data from the KM plotter database, including 504 late-stage LUAD and 1,262 late-stage BRCA patients, were used. The median miR-29b-3p expression was used as a cutoff value. It was found that the miR-29b-3p high expression group had a higher survival probability than the low expression group, which was 58 months in LUAD (vs. 42 months for low group, *P* = 0.012, log-rank test, Figure 2A) and 198 months in BRCA (vs. 180 months for low group, *P* = 0.0050, log-rank test, Figure 2B). These data suggested that the high expression of miR-29b-3p had significant advantages in cancer treatment.

Previous research reported that MYC Proto-Oncogene (c-MYC) directly suppresses miR-29b-3p (Chang et al., 2008). MiR-29b-3p is known to critically affect cancer progression by functioning as a tumor suppressor (Yan et al., 2015), and it targets several DNA methyltransferases (DNMTs, including DNMT1, DNMT3B, etc.) and regulates members of the DNA demethylation signaling pathway, leading to the downregulation of global DNA methylation in malignant cells (Garzon et al., 2009b). In addition, miR-29b-3p inhibits cell proliferation and promotes apoptosis by targeting B-cell lymphoma 2 (Bcl-2), phosphoinositide-3-kinase regulatory subunit 1 (PIK3R1), and AKT serine/threonine kinase 2 (AKT2) (Figure 2C). Because the miR-29b-3p expression is different between 3D and 2D cultured cells, we speculated that the expression of c-MYC and miR-29b-3p downstream oncogenes could be different as well. Figure 2D shows that the expression of the genes mentioned earlier was higher in unirradiated A549 cells cultured in 3D than in those of 2D culture. At the time point of 1, 6, and 12 h after 5-Gy X-ray irradiation, c-MYC, DNMT3B, PIK3R1, AKT2, and Bcl-2 maintained high expression in 3D cells. These results indicated that high expression of c-MYC suppresses miR-29b-3p and increases expression of DNMT3B, PIK3R1, AKT2, and Bcl-2. Recent reports have demonstrated that c-MYC contributes to chemotherapeutic resistance in various CSCs (Elbadawy et al., 2019; Zhang et al., 2019), and CSCs are generally resistant to conventional chemotherapy and radiotherapy through activation of DNMT1, DNMT3B, Bcl-2, and cellular pro-survival signaling pathways PI3K/AKT (Berghauser Pont et al., 2014; Wang et al., 2014; Liu et al., 2015; Xia and Xu, 2015; Chang et al., 2016; Zagorac et al., 2016). These results suggested that c-MYC/miR-29b-3p and downstream DNMT3B, Bcl-2, and PIK3R1/AKT2 may be involved in radioresistance in 3D cultured cells.

### Knockdown of miR-29b-3p Enhances Radioresistance of Two-Dimensional Cultured A549 and MCF7 Cells

**FIGURE 3:**
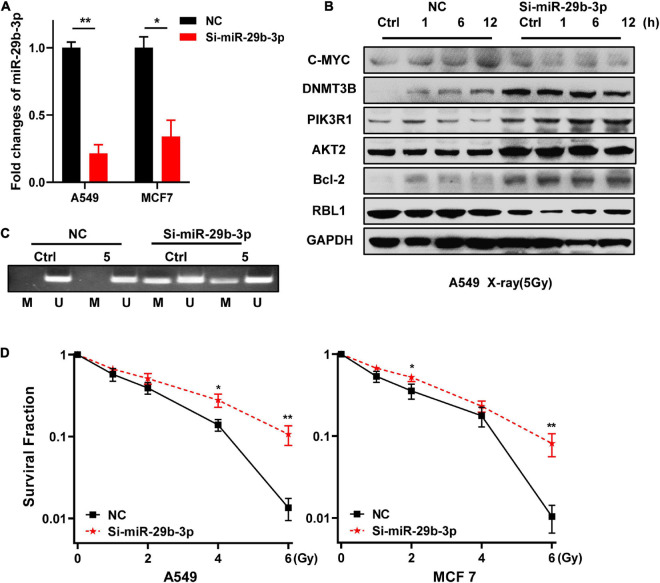
Knockdown of miR-29b-3p enhances the radioresistance of 2D cultured A549 and MCF7 cells by regulating oncogenes. **(A)** Relative expression levels of miR-29b-3p measured by qRT-PCR in 2D miR-29b-3p knockdown A549 and MCF7 cells. **(B)** Western blot assay on the expression of c-MYC, DNMT3B, PIK3R1, AKT2, Bcl-2, and RBL1 at indicated time points after 5 Gy X-rays in 2D cultured miR-29b-3p knockdown A549 cells. **(C)** The changes of the promoter methylation status of RBL1 measured by MSP in 5 Gy X-ray irradiated and unirradiated 2D cultured miR-29b-3p knockdown A549 cells. **(D)** Colony formation assay on the 2D cultured miR-29b-3p knockdown A549 and MCF7 cells after exposure to 0, 1, 2, 4 and 6 Gy X-rays. Ctrl, unirradiated groups. NC: negative control. Significance was determined by unpaired *t*-test. *P < 0.05; **P < 0.01.

Because high expression of miR-29b-3p benefits the prognosis of LUAD and BRCA, we investigated whether miR-29b-3p was involved in the radioresistance. Firstly, we constructed miR-29b-3p knockdown stable cell lines in 2D cultured A549 and MCF7 cells by lentivirus. As shown in Figure 3A, the expression of miR-29b-3p significantly decreased in miR-29b-3p knockdown cell lines. In the miR-29b-3p knockdown groups, the expression of DNMT3B, PIK3R1, AKT2, and Bcl-2 was maintained at a high level after 5-Gy X-ray radiation. The miR-29b-3p level had no impact on c-MYC expression (Figure 3B). Our previous study revealed that promoter methylation of transcriptional corepressor like 1 (RBL1) enhances the radioresistance of 3D cells (Pan et al., 2016a). We detected the correlation between miR-29b-3p and RBL1 promoter methylation. The results show that a low level of miR-29b-3p decreased RBL1 expression (Figure 3B) and methylated promoter region of RBL1 in 2D cultured A549 cells. The promoter of RBL1 maintained the methylated status after 48 h in 5-Gy X-ray irradiated cells (Figure 3C), which accounts for the RBL1 high expression in the miR-29b-3p knockdown group. Figure 3D showed that in the miR-29b-3p knockdown groups, survival fractions of X-ray irradiated 2D cultured A549 and MCF7 cells were higher than those of negative control. These results suggested that the knockdown of miR-29b-3p enhances the radioresistance of 2D cultured cells.

### Overexpression of miR-29b-3p Sensitizes the Three-Dimensional Cultured A549 and MCF7 Cells to Radiation

**FIGURE 4:**
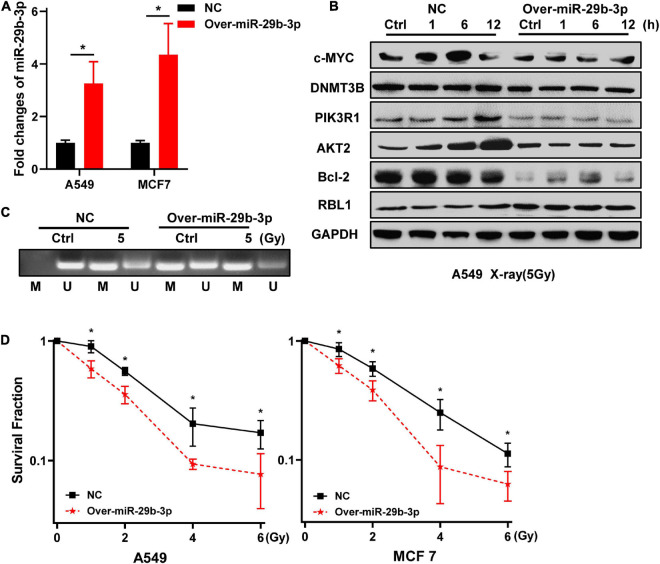
Overexpression of miR-29b-3p sensitizes the 3D cultured A549 and MCF7 cells to radiation. **(A)** Relative expression of miR-29b-3p measured by qRT-PCR in 3D culture miR-29b-3p overexpressed A549 and MCF7 cells. **(B)** Western blot assay on the expression of c-MYC, DNMT3B, PIK3R1, AKT2, Bcl-2 and RBL1 at indicated time points after 5 Gy X-rays in 3D cultured miR-29b-3p overexpressed A549 cells. **(C)** The changes of the promoter methylation status of RBL1 measured by MSP in 5 Gy X-ray irradiated and unirradiated 3D cultured miR-29b-3p overexpressed A549 cells. **(D)** Colony formation assay on the 3D cultured miR-29b-3p overexpressed A549 and MCF7 cells after exposure to 0, 1, 2, 4 and 6 Gy X-rays. Ctrl, unirradiated groups; NC, negative control. Significance was determined by unpaired *t*-test. *P < 0.05.

Because knockdown of miR-29b-3p enhanced the radioresistance of 2D cultured A549 and MCF7 cells, we next examine whether the overexpression of miR-29b-3p decreases the radioresistance of 3D cells. As shown in Figure 4A, miR-29b-3p expression significantly increased in 3D cultured A549 and MCF7 cells infected with lentivirus containing miR-29b-3p overexpression vector, compared with the negative control groups. In the miR-29b-3p overexpression groups, DNMT3B, PIK3R1, AKT2, and Bcl-2 expression levels were lower than that of negative control groups after 5-Gy X-ray radiation. Meanwhile, RBL1 expression increased in the miR-29b-3p overexpressed groups, but c-MYC expression had no obvious change (Figure 4B). Figure 4C showed that the high level miR-29b-3p demethylated promoter region of RBL1 and resulted in RBL1 high expression in miR-29b-3p overexpressed groups. This is because the survival fractions of miR-29b-3p overexpressed A549 and MCF7 cells in 3D culture exposed to X-rays were lower than those of the negative control, suggesting that overexpression of miR-29b-3p sensitizes 3D cultured A549 and MCF7 cells to radiation (Figure 4D).

### Overexpression of miR-29b-3p Increases Tumor Radiosensitivity *in vivo*

**FIGURE 5:**
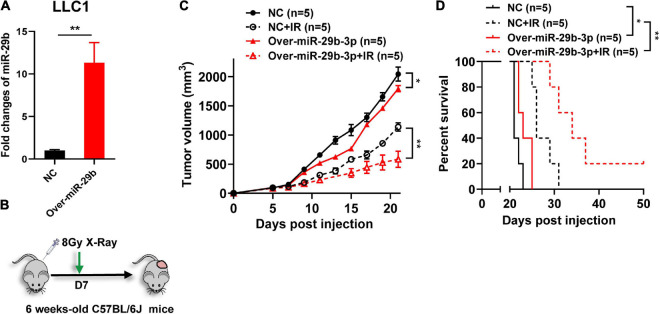
The miR-29b-3p expression level on the efficacy of radiotherapy *in vivo*. **(A)** Relative expression of miR-29b-3p measured by qRT-PCR in miR-29b-3p overexpressed LLC1 cells. **(B)** Protocol for radiation treatment. **(C,D)** Tumor growth and Kaplan–Meier survival curves of C57BL/6 mice inoculated with 2 × 105 VC or miR-29b-3p overexpressed LLC1 cells. Radiotherapy was conducted lx at 8 Gy at 7 days post inoculation of the tumor cells. NC, negative control. Significance was determined by 2-way ANOVA in c, logrank test in d respectively. *P < 0.05; **P < 0.01.

To further investigate the effect of miR-29b-3p on radiosensitivity *in vivo*, mice were injected with the miR-29b-3p overexpression or vector control LLC1 cells and exposed to 8-Gy X-ray after 7 days (Figures 5A,B). Overexpression miR-29b-3p resulted in significant inhibition on tumor growth and prolonged host survival in the lung cancer mouse model, with one of five mice had been cured (Figures 5C,D). These results suggested that overexpression of miR-29b-3p significantly enhances radiosensitivity *in vivo*.

### miR-29b-3p Interferes With the Kinetic Process of DNA Damage Repair in A549 Cells

**FIGURE 6:**
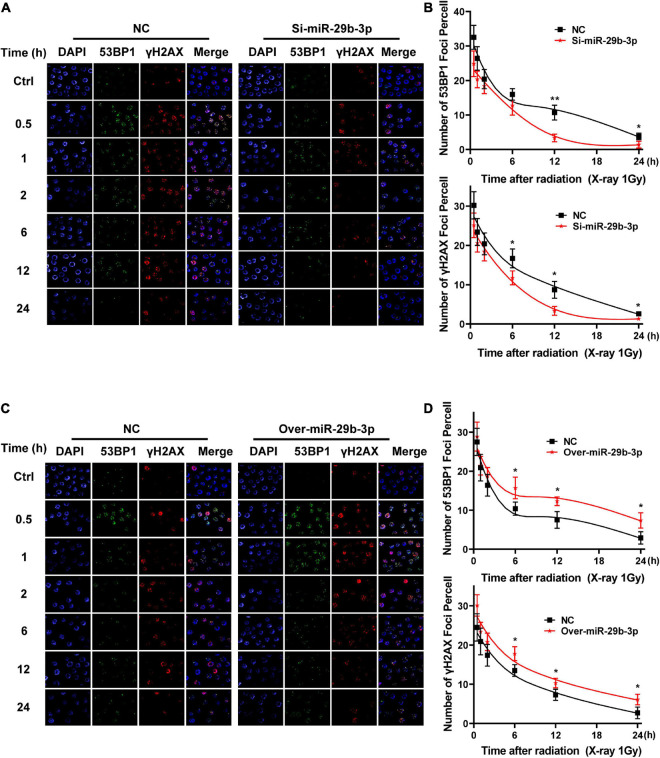
Kinetics process of DNA damage repair in miR-29b-3p knockdown or overexpressed A549 cells. **(A)** Graph shows quantification of 53BP1 and yH2AX foci in miR-29b-3p knockdown and negative control A549 cells exposed to 1 Gy X-ray. **(B)** The numbers of 53BP1 and yH2AX foci in 50 cells of each group were counted for each time point. **(C)** Graph shows quantification of 53BP1 and yH2AX foci in miR-29b-3p overexpressed and negative control A549 cells exposed to 1 Gy X-ray. **(D)** The numbers of 53BP1 and yH2AX foci in 50 cells of each group were counted for each time point. Ctrl, unirradiated groups; NC, negative control. Significance was determined by unpaired *t*-test. *P < 0.05; **P < 0.01.

We have demonstrated that miR-29b-3p influences cancer cells’ radiosensitivity; we further investigated whether miR-29b-3p impacts the kinetic process of DNA damage repair. We examined the 53BP1 and γH2AX foci that are surrogate markers of DNA damage repair by immunofluorescence staining in the 2D cultured A549 cells. In 1-Gy X-ray irradiated miR-29b-3p knockdown A549 cells, the kinetics of foci dissolution of 53BP1 and γH2AX were faster than in negative groups after 12 h (Figures 6A,B). Moreover, more 53BP1 and γH2AX foci remained in miR-29b-3p overexpressed A549 cells than in negative control cells after 6 h (Figures 6C,D). These results indicated that miR-29b-3p impacts the recruitment of the DNA repair complex.

### Knockdown of miR-29b-3p Rescues the Invasion and Migratory Capacity of Irradiated A549 and MCF7 Cells

**FIGURE 7:**
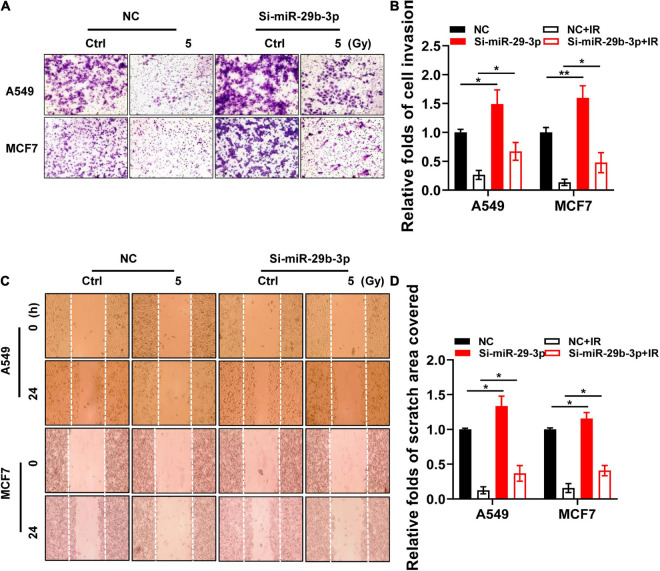
Invasive and migratory capacity of irradiated nniR-29b-3p knockdown A549 and MCF7 cells. **(A,B)** Matrigel invasion assays in miR-29b-3p knockdown or negative control A549 and MCF7 cells after 5 Gy X-ray irradiation at 0 and 48 h. **(C,D)** Scratch wound healing migration assays in miR-29b-3p knockdown or negative control A549 and MCF7 cells after 5 Gy X-ray irradiation at 0 h and 24 h. Ctrl, unirradiated groups; NC, negative control. Significance was determined by unpaired *t*-test. *P < 0.05; **P < 0.01.

Epithelial–mesenchymal transition (EMT) is a critical step in cancer cell invasion and metastasis, and it positively correlates with poor patient prognosis. In addition to its roles in cell proliferation, apoptosis, and differentiation, miR-29b-3p also exerts effects on cell migration and invasion *in vitro* by regulating EMT signaling (Ru et al., 2012; Kinoshita et al., 2013; Poudyal et al., 2013). According to the pieces of evidence discussed earlier, we hypothesized that miR-29b-3p knockdown might rescue the migratory and invasive capacity of irradiated cancer cells. Matrigel invasion assay showed that miR-29b-3p knockdown A549 and MCF7 cells exhibited more marginal invasion through the extracellular matrix after 48 h in both irradiated and unirradiated cells compared with control cells (Figures 7A,B). Figures 7C,D show that miR-29b-3p knockdown A549 and MCF7 cells had a remarkable ability on promoting cell migration in the scratch wound after 24 h in both irradiated and unirradiated cells compared with control cells. Thus, the effects of miR-29b-3p on cell migration and invasive properties may play a critical role in poor patient prognosis with radiation therapy.

### miR-29b-3p Is Associated With Stemness and Oncogene Expression in Cancer Stem Cells

**FIGURE 8:**
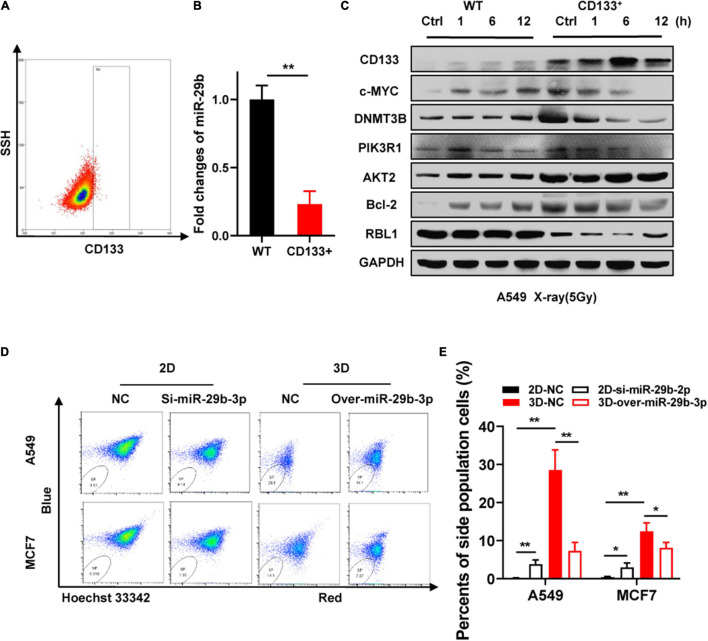
Correlation between miR-29b-3p expression and cancer cell sternness. **(A)** Sorting CD133+ A549 cells by flow cytometry. **(B)** Relative miR-29b-3p expression measured by qRT-PCR in CD133+ and negative control A549 cells. **(C)** Western blot assay on the expression of CD133, c-MYC, DNMT3B, PIK3R1, AKT2, Bcl-2 and RBL1 at indicated time points after 5 Gy X-rays in CD133+ A549 cells and negative control cells. **(D,E)** Side population in miR-29b-3p knockdown 2D and overexpressed 3D culture A549 cells. Ctrl, unirradiated groups; NC, negative control; WT, wild type groups. Significance was determined by unpaired *t*-test. *P < 0.05; **P < 0.01.

CD133 is currently considered the most robust surface marker for CSCs in various tumor types (Glumac and LeBeau, 2018). Wang et al. (2015) reported low expression of miR-29b in CD133-positive A549 cells. MiR-29b-3p is significantly downregulated in CD133 + cells separated from the peripheral blood of HCC patients (Zekri et al., 2018). As our previous study revealed that 3D cultured cells simulate some stem cell characteristics and have high radioresistance (Xue et al., 2015b), we speculated that miR-29b-3p deficiency in 3D cells might account for these phenomena. Flow cytometry was used for CD133-positive A549 cell sorting (Figure 8A). Figure 8B shows a significantly low expression of miR-29b-3p in CD133-positive A549 cells by qRT-PCR. In the CD133-positive A549 cells, cMYC, RBL1, and oncogene expression at indicated time points after 5-Gy X-rays have a similar tendency with 3D cultured cells (Figure 8C). Hoechst 33342 side population (SP) analysis is a common method for identifying stem cells in mammalian tissues (Telford et al., 2007). Flow cytometry analysis shows that the number of SP cell proportions was higher in 3D cultured A549 and MCF7 cells compared with 2D cells. MiR-29b-3p knockdown in 2D cells could increase the SP cell proportion. Inversely, miR-29b-3p overexpression in 3D cells would alleviate the SP cell proportion (Figures 8D,E). These results demonstrate that miR-29b-3p is associated with stemness characters and oncogenes PIK3R1, AKT2, and Bcl-2 expression in CSC.

## Discussion

Lung cancer is one of the most frequently diagnosed cancers in both incidence and mortality, and breast cancer is the world’s most prevalent cancer in women (Smith et al., 2019). Radiotherapy is one of the main modalities in lung and breast cancers, but one of the main obstacles is these tumor types exhibit significant radioresistance. However, cancer radioresistance can result in tumor recurrence and contributes to the poor prognosis of cancer patients. Thus, there is a great interest in understanding the underlying biology and developing strategies to overcome this problem. The mechanisms underlying the development of radioresistance, which are involved in multiple genes and factors, have been the focus of many studies (Tang et al., 2018). CSCs is one of the important reasons for cancer resistance to chemotherapeutic agents and radiation therapy (Phillips et al., 2006; Blazek et al., 2007; de Jong et al., 2010; Li et al., 2016). Characterizing the roles of CSCs in radioresistance and identifying the molecular pathways that maintain CSC stemness are of paramount importance in improving the efficacy of cancer treatments.

Deregulation of various epigenetic regulations such as DNA methylation, histone modification, and miRNAs can contribute to tumor initiation and tumorigenesis, particularly concerning the maintenance and survival of CSCs (Toh et al., 2017). Importance of aberrant miRNA expression levels in maintaining CSC properties have been reported in CSCs (Lu et al., 2005). MiRNAs offer new possibilities for targeting CSCs as well as improving overall cancer therapy.

The 3D cultured model mimics *in vivo* microenvironment and has numerous different characteristics compared with 2D culture, which is a convenient method to induce CSC-like properties (Chen et al., 2012; Xue et al., 2015b; Usui et al., 2016). The combinatorial effects of epigenetic regulation and ionizing radiation contribute to eliminating CSCs have been reported (Cui et al., 2014; Peitzsch et al., 2016; Kwon et al., 2017). MiRNAs showed differential expression profiles between 2D and 3D cells, and miR-29b-3p had the most dramatic negative change in cells of 3D compared with 2D cultures. MiR-29b-3p is a member of the miR-29 family, which includes miR-29a, b, and c. Recently, numerous studies have demonstrated that aberrant expression of miR-29b-3p is common in the majority of human cancers (Garzon et al., 2009a; Zhang et al., 2011; Kriegel et al., 2012; Rossi et al., 2013). MiR-29b-3p is known to critically affect tumorigenicity and stemness maintenance by functioning as a tumor suppressor (Wang et al., 2015; Shin et al., 2017; Zhang et al., 2018). Lee et al. reported that miR-29b-3p suppressed by c-Myc in lung cancer patients led to significantly worse survival outcomes (Wu et al., 2015). Analysis from The Cancer Genome Atlas clinical data indicated that miR-29b-3p contributed to better prognosis in LUAD and BRCA patients. We speculated that miR-29b-3p is involved in radiation response and effected radioresistance in stem-like 3D cultured cancer cells. cMYC represses miR-29b-3p directly and has higher expression in 3D compared with 2D cultured cells (Chang et al., 2008), which might account for the low miR-29b-3p expression in 3D cells. Emerging evidence suggested that miR-29b-3p could serve as a tumor suppressor gene by targeting DNMT3B, Bcl-2, PI3KR1, and AKT2 (Garzon et al., 2009b; Park et al., 2009; Xiong et al., 2010). Previous research reported that DNM3B deficiency radiosensitizes by RBL1 or disrupts DNA damage regulation (Fujimori et al., 2015; Pan et al., 2016a). Meanwhile, the lung cancer patients treated with radiotherapy had poorer survival in Bcl-2 overexpressing group than patients without Bcl-2 expression (Hwang et al., 2001). In addition, suppressing PI3K/AKT2 signaling pathway potentiated the irradiation effect by mediated DNA repair both *in vivo* and *in vitro* (Toulany and Rodemann, 2013; Tang et al., 2015). Our subsequent experiments demonstrated that low expression of miR-29b-3p in 3D cultured cells resisted radiation killing and had a change of the expression of DNMT3B, Bcl-2, PI3KR1, AKT2, and RBL1. Overexpression of miR-29b-3p significantly enhanced radiosensitivity both *in vitro* and *in vivo* and inhibited the kinetic process of DNA damage repair followed by altering expression of DNMT3B, Bcl-2, PI3KR1, AKT2, and RBL1. The phenomenon mentioned earlier can be repeated in CD133^+^ stem-like cells.

CSCs are key drivers of tumor progression that promote migration and invasion *via* induction of EMT, leading to metastasis and tumor recurrence (Ayob and Ramasamy, 2018). Increasing evidence indicated that miR-29b-3p also exerted effects on cell migration and invasion (Ru et al., 2012; Kinoshita et al., 2013; Poudyal et al., 2013). Our results showed miR-29b-3p deficiency could rescue the invasion and migratory capacity of irradiated tumor cells, which would enhance the ability of radiation killing and eradicate metastasis and tumor recurrence. Furthermore, miR-29b-3p deficiency augments SP cell proportions in 2D cultured cells, indicating tumor stemness.

In conclusion, our study suggested that miR-29b-3p could influence DNA damage response by regulating the expression of DNMT3B, Bcl-2, PI3KR1, AKT2, and RBL1, thereby affecting tumor radioresistance. MiR-29b-3p assisted/conjugated therapies could have greater potential to overcome radiotherapy resistance in cancers.

## Data Availability Statement

The data presented in the study are deposited in the NCBI BioProject, accession number (PRJNA756383).

## Ethics Statement

The animal study was reviewed and approved by Wenzhou Medical University Institutional Animal Use and Care Committee.

## Author Contributions

BH and DP contributed to conception and design of the study and wrote the first draft of the manuscript. DP, YD, RL, and AS performed the investigation. YD, XL, and CL wrote sections and reviewed the manuscript. All authors contributed to manuscript revision, read, and approved the submitted version.

## Conflict of Interest

The authors declare that the research was conducted in the absence of any commercial or financial relationships that could be construed as a potential conflict of interest.

## Publisher’s Note

All claims expressed in this article are solely those of the authors and do not necessarily represent those of their affiliated organizations, or those of the publisher, the editors and the reviewers. Any product that may be evaluated in this article, or claim that may be made by its manufacturer, is not guaranteed or endorsed by the publisher.

## Footnotes

1
https://kmplot.com/analysis/
